# Nanostructured versus flat compact electrode for triboelectric nanogenerators at high humidity

**DOI:** 10.1038/s41598-021-95621-3

**Published:** 2021-08-10

**Authors:** Masoume Karimi, Sadegh Seddighi, Raheleh Mohammadpour

**Affiliations:** 1 Department of Mechanical Engineering, K. N. Toosi University of Technology, 19919-43344 Tehran, Iran; 2 Institute for Nanoscience and Nanotechnology, Sharif University of Technology, 14588-89694 Tehran, Iran

**Keywords:** Energy science and technology, Engineering

## Abstract

The triboelectric nanogenerator (TENG) is a promising technology for mechanical energy harvesting. TENG has proven to be an excellent option for power generation but typically TENGs output power drops significantly in humid environments. In this work, the effect of electrode’s material on power output, considering smooth and nanostructured porous structures with various surface hydrophobicity, is investigated under various humidity conditions. A vertical contact-separation mode TENG is experimentally and numerically studied for four surface morphologies of Ti foil, TiO_2_ thin film, TiO_2_ nanoparticulated film, and TiO_2_ nanotubular electrodes. The results show that the TENG electrical output in the flat structures such as Ti foil and TiO_2_ thin film at 50% RH is reduced to 50% of its initial state, while in the nanoporous structures such as nanoparticle and nanotube arrays, this is observed at RH above 95%. The results show that the use of porous nanostructures in TENG due to their high surface-to-volume, and that the process of water adsorption on the pore leads to better performance than the flat surface in humid environments. Based on our study, employing nanoporous layers is vital for nanogenerators either for power generation or active sensor applications at high humidity conditions.

## Introduction

Limitation of fossil fuel resources and their related environmental impacts caused a sharp increase in demands for alternative energy sources in recent years. There has been a great tendency toward the development of novel technologies for extracting energy from clean and renewable resources such as water, wind, solar, ocean and geothermal^1,2^. Among different energy sources, mechanical energy is available widely in our environment^3^. Human kinetic energy such as breathing, heart beating, running, talking, touching, typing, and also environmental mechanical energy sources such as wind, water, and tire rotation are some of the accessible mechanical energy sources^4–9^. There are several methods to convert mechanical energy to electricity such as electromagnetic, piezoelectric and triboelectric nanogenerator^10,11^. Among these technologies, triboelectric nanogenerators have attracted attention since they are eco-friendly, cost-effective, efficient, lightweight, simple, and easy to fabricate^12–15^. In addition, TENG can employ energy from low-frequency external vibrations. This unique feature is used to fabricate the self-powered applications that depend on environmental mechanical energy as their power source^16^. TENG has diverse applications such as smart wearable devices, self-powered devices, health sensors, and environmental sensors for monitoring variations in pressure, temperature and humidity^15,17–20^.

The mechanism principle of TENG is based on triboelectrification contact and electrostatic induction^19,21,22^. In the triboelectrification phenomenon, contact between two materials with different triboelectric affinity produces a charge and after surfaces separate from each other, the electrostatic induction causes the flow of electron current between two electrodes^4,19,23,24^. TENG performance is affected by the charge generation^25^. The charge generation depends on triboelectric materials, surface morphology, and environmental factors such as humidity^5,16,26–29^. By selecting materials with a large different polarity, the TENG surface charge density increases^12,13^. Improved surface morphology can enhance the effective contact area^30^. In a flat surface, the amount of the nominal contact area is equal to the real contact area but in a rough or porous structure applying a mechanical force deform these surfaces forcing them to fill vacant spaces due to the elastic property, leading to an increased effective surface area^31^. So covering the substrate surface with the nanotube, nanoparticle, nanowire, and porous media can improve the performance of TENG due to an increased internal surface area as also suggested in^13,15,32–35^. On the other hand, TENG charge vanishes in humid environments because of the formation of water film on the surface^19,36,37^. Since humidity is ubiquitous in environments where TENG is used, improving the TENG operation in humid environments is of great importance as also indicated in^38,39^.

Among different materials proposed for the TENG in humid environments, Titanium dioxide (TiO_2_ metal-oxide) attracted attention because of its properties. For instance, TiO_2_ is a stable material and can be synthesized with various morphologies where it has high surface-to-volume in TiO_2_ nanostructure. Thus, TiO_2_ is used widely in diverse applications such as in active self-powered sensors, UV-photodetectors, and biosensors^40,41^. TiO_2_ which is a superior semiconductor is extensively utilized in various applications such as in different types of gas, bio and chemical sensors, photovoltaic cells, photoelectrochemical cells, photocatalysis, and anti-corrosion surfaces^42^. On the other hand, TiO_2_ has been considered as one of the most positively charged metal oxides in the triboelectric series followed by Al_2_O_3_, SiO_2_, and HfO_2_^43^. In this study, a triboelectric nanogenerator based on TiO_2_ as the positive triboelectric material is proposed taking advantage of the superior feature of TiO_2_ and the possibility of energy production by this material. On the other hand, Polyimides such as the Kapton layer has been extensively employed in TENG modules as a negative friction layer due to outstanding stability and mechanical feature along with it’s negative releasing (or positive pressing) nature^44–47^. This type of TENG will have a potential application as a self-powered gas sensor and photodetector. In addition, the hydrophilicity of the TiO_2_ layer is extensively influenced by surface morphology where the wettability of either TiO_2_ surface could be reversible between hydrophobicity and hydrophilicity^42^. Therefore, to investigate the effect of wettability on TENG performance, TiO_2_ can be considered as the suitable candidate.

Surface morphology is very important for TENGs performance due to the impacts of interfacial phenomena on the charge^33^. To improve the surface morphology, Huang et al.^48^ have fabricated a TENG enhanced by micro/nano structures using femtosecond laser direct writing method and achieved 21 times enhancement in power density compared to a flat-structured TENG. Muthu et al.^49^ introduced a TENG modified by laser surface patterning technique to form micro patterns such as circle, line and X patterns to enhance the surface contact area and showed that line pattern was the best choice. Feng et al.^50^ used a polypropylene nanowire array modified by fluorinated compounds in TENG layers and achieved 4 times enhancement in the electrical output compared to a flat surface. Several studies have been conducted on the TENG in the presence of humidity in recent years. Gue et al.^51^ introduced a TENG to monitor humidity and airflow rate. In their study, output current was used to measure the various environmental conditions and approaching zero at high RH. Su et al.^52^ proposed a combination of TENG with a resistive humidity sensor to detect moisture that has a good response/recovery time and stability but the voltage dropped significantly at high RH. Ma et al.^53^ fabricated an active multifunctional sensor based on polytetrafluoroethylene (PTFE) and Aluminum foil that measured humidity, airflow rate, and motion. In their design, by increasing the RH, the output voltage decreased so that in 100% RH the output approached zero. Jao et al.^54^ fabricated a textile-based self-powered humidity and sweat sensor by combining a chitosan-based humidity resistor and TENG that had properties such as flexibility, transparency, and biocompatibility. In their study, relative humidity changed from 20 to 80% and the output dropped linearly by increasing the RH. Zhang et al.^55^ designed a humidity sensor based on tin disulfide nano flowers/reduced graphene oxide (SnS_2_/RGO) which had flexibility, stability, high power, and also good response/recovery time, but in the high-humid ambient (over to 75%) the voltage output almost vanished. In all these studies, the TENG performance dramatically decreases at high relative humidity so the output current and voltage at RH above 80% tend to be almost zero which limits TENG applicability in moisture ambient as a self-power application and humidity sensors. Therefore, the design and fabrication of a TENG that could maintain its quality and electrical output at high relative humidity is still a challenge.

To solve the TENG efficiency drop at increased humidity, this work experimentally and numerically improves the TENG performance by choosing the best morphologies and improving surface properties. The novelty of this work lies in improved TENG electrode structures capable of keeping designed electrical output at high relative humidity (up to 95% RH). The aim of this study is to (1) specify which of the nanostructured (including TiO_2_ nanoparticles and TiO_2_ nanotube arrays) or flat compact (including Ti foil and TiO_2_ thin film) electrodes is the most suitable for TENG operation at a wide range of humidities, (2) investigate the effect of using the porous structure compared to a flat surface in the process of water adsorption and the corresponding induced charge in a humid environment and (3) study the effect of dielectric-dielectric and metal-dielectric structure and dielectric constant in the TENG. To reach this goal, four morphologies including Ti foil, TiO_2_ thin film, TiO_2_ nanoparticulate, and TiO_2_ nanotubes were selected as the active electrodes in TENG, and the electrical outputs were investigated experimentally and numerically. Firstly, an experimental module of TENG is fabricated for four morphologies and then during the experiments, the output current, voltage, and charge transfer of TENG are measured. In the next step, this TENG module is simulated numerically by using the main governing equations of the contact-separation mode of TENG.

## Experimental setup

**Figure 1:**
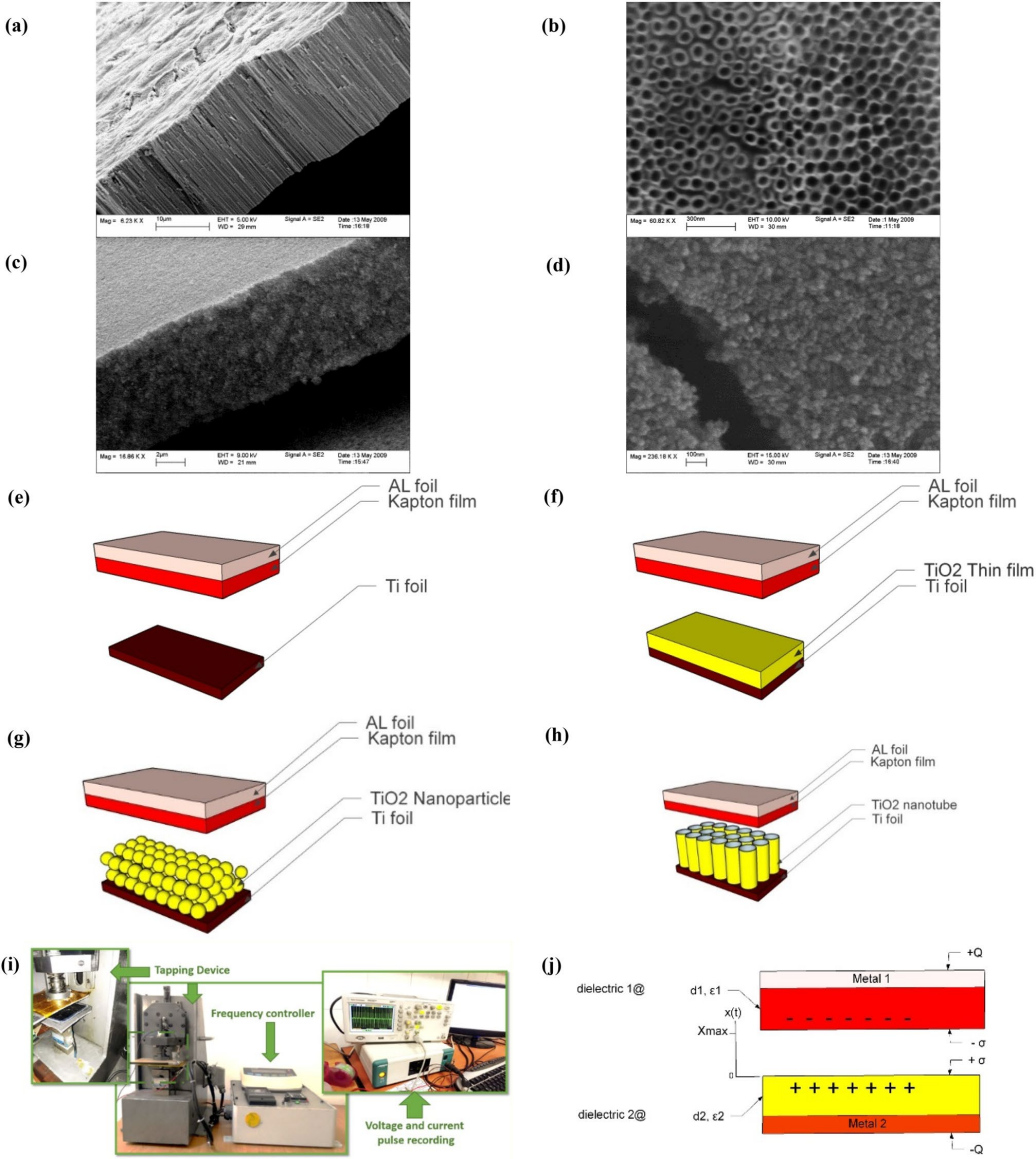
The structural design of the TENG based on the different morphology in the triboelectric active layer. (**a,b**) SEM images of TiO_2_ nanotube film and (**c,d**) TiO_2_ nanoparticle film. The structure of fabricated TENG based on different active layers: (**e**) Ti foil, (**f**) TiO_2_ thin film, (**g**) TiO_2_ nanoparticulate film, and (**h**) TiO_2_ nanotube array film. (**i**) Experimental setup. (**j**) Schematic diagram of TENG in numerical simulation and its related parameters.

For a better understanding of the effect of surface morphology on the TENG performance in a high humidity environment, two types of structures consist of the flat surface including Ti foil and TiO_2_ thin film and nanostructured surface including TiO_2_ nanoparticles and TiO_2_ nanotube arrays have been employed as the triboelectric active layer of TENG. The compact layer of TiO_2_ was fabricated by annealing Ti foil at 450 °C for 12 h. TiO_2_ nanotube arrays fabricated through two-electrode anodization of 250 µm Titanium foil (99.5%; Alfa Aesar) at 60 V bias in the organic electrolyte containing ethylene glycol with the addition of 0.2 vol % H_2_O and 0.3 M NH_4_F followed by annealing at 450 °C for 6 h in pure. As it is illustrated in the SEM image of Fig. 1a,b, nanotubes have an average length of 20 µm with an outer diameter of 100 nm and a wall thickness of 15 nm. Nanoparticulated films of 20 µm TiO_2_ have been fabricated by Doctor-blading commercial TiO_2_ paste (Sharif Solar) followed by annealing at 450 °C. The SEM imaged of nanoparticle film is shown in Fig. 1c,d.

A vertical contact-separation mode TENG is employed in this research. Kapton film with a thickness of 20 μm and an area of 2 cm × 2 cm was selected as the fixed part of TENG in all experimental setups. In the first case, Ti foil was selected as the active electrode and made metal-to-dielectric TENG, as illustrated in Fig. 1e. In the second case, a TiO_2_ thin film with a thickness of 500 nm was grown on Ti foil, as the moving part of TENG (Fig. 1f). In the third case, the TiO_2_ nanoparticulated film with a thickness of 20 μm and a particle diameter of about 20 nm was fabricated on the Ti foil as the active triboelectric layer (Fig. 1g). In the fourth case, the TiO_2_ nanotubes were prepared as the triboelectric active layer, as shown in Fig. 1h.

The test setup has been illustrated in Fig. 1i. The fixed part and moving parts of the TENG are attached to the Aluminum tape as the back-contact in the tapping device. Electrodes were mounted on the suitable holders on the tapping device. The tapping device, the pipe that is used to transport water vapor, and the aperture for wires are surrounded by a plastic chamber. All electrical outputs were measured under the periodical compressive force of 8.3 N, at the vibration frequency of 2 Hz and the gap distance of 2 cm. SEM images were recorded with the Zeiss (EM10C-80 kV) and Tescan (MIRA3) electron microscope. The output voltage and current were recorded by an oscilloscope (DSO1022A) and the Ivium Compactstat, respectively. The humidification for measuring the sensor response at different relative humidity values was achieved by using a humidifier to regulate the ambient humidity that can change the RH from 20 to 99%. Also, a commercial hygrometer-temperature sensor was used to monitor the amount of RH during the experiment. The surface hydrophobicity and the contact angle measurements were carried out using instrumnet Dataphysics-OCA 15 plus. A drop of water (4 μl) was placed on samples and the image was immediately sent via the CCD camera. The contact angle tests were performed under 3006-0102-7 standard condition.

## Numerical simulation

A mathematical two-dimensional model is developed to simulate the vertical mode TENG and the electrical outputs are derived by utilizing the Gauss theorem considering the structure of the solid system at the electronic structure level^56^. Theoretical modeling of TENG was carried out based on Maxwell's theory^56^ where current and voltage are predicted by incorporating mathematical modeling. Since different environmental parameters such as humidity, have an impact on the physical properties of a porous layer such as dielectric constant, a model based on Bruggeman's theory is developed to predict the effective dielectric constant influenced by humidity in the original TENG equation^57^. The resulting model enables capturing the physical phenomena behind water penetration inside pores which results in losing surface charges. A simplified 2-D geometry contact-separation mode of TENG and its related parameters that are used in the numerical simulation is shown in Fig. 1j. Based on Fig. 1j, the two dielectric layers with thicknesses of *d*_*1*_ and *d*_*2*_ and the relative dielectric constants *ε*_*r1*_ and *ε*_*r2*_ are attached on metal electrodes and construct the triboelectric pair. The distance between the two triboelectric pairs is *x*(*t*) and can change under external mechanical force. $\sigma$ is the surface charge density created by the electrification phenomenon, and *Q* is defined as the transferred charge between the two electrodes based on electrostatic induction.

The edge effect can be negligible since the surface area is much larger than the distance between the two electrodes^56^. The electric field inside the dielectric 1, 2, and air gap is given by Gauss' law^58^:

1 $${E}_{1}=- \frac{Q}{S{\varepsilon }_{0}{\varepsilon }_{r1}}$$

2 $${E}_{\mathrm{air}}= \frac{-\frac{Q}{S}+ \sigma }{{\varepsilon }_{0}}$$

3 $${E}_{2}=- \frac{Q}{S{\varepsilon }_{0}{\varepsilon }_{r2}}$$

where *E* (N/C) is the electrical field, *Q*(C) is induced charge, $\sigma$ (N/m^2^) is surface charge density, *S*(m^2^) is surface area, *ε*_*r*_ is the relative dielectric constant and *ε*_*0*_ (C^2^/N.m^2^) is vacuum permittivity.

The voltage differences between the two electrode metals can be given by Eqs. (4) and (5) according to^58,59^:

4 $$V=\int E\cdot dn$$

5 $$V={E}_{1}{d}_{1}+{E}_{2}{d}_{2}+{E}_{air}x(t)$$

where *n* is the coordinate perpendicular to the surface. *V*(v) is the output voltage. *x*(m) is the distance between the two triboelectric pairs that changes by the mechanical force which is shown in Eq. (6):

6 $$x(t)={x}_{Max} \left (\frac{1}{2}-\frac{1}{2} \left (\mathrm{cos} \left(\frac{2\pi f}{{x}_{Max}}t \right) \right) \right)$$

where *x*_*max*_(m) is the maximum distance between two dielectric and *f*(Hz) is the moving frequency.

By substituting Eqs. (1)–(3) into Eq. (5), the output voltage can be obtained as a function of *Q*, *x*, *d*, *ε*_*r*_, and *t* by Eq. (7)^58^:

7 $$V=-\frac{Q}{S{\varepsilon }_{0}}\left(\frac{{d}_{1}}{{\varepsilon }_{r1}}+\frac{{d}_{2}}{{\varepsilon }_{r2}}+x\left(t\right)\right)+\frac{\sigma x\left(t\right)}{{\varepsilon }_{0}}$$

And the effective thickness defines by Eq. (8):

8 $${d}_{0}=\frac{{d}_{1}}{{\varepsilon }_{r1}}+\frac{{d}_{2}}{{\varepsilon }_{r2}}$$

In the short circuit (SC) condition, *V* is equal to 0 in Eq. (7). Therefore, the short circuit transferred charge and current are given by Eqs. (9) and (10)^58^:

9 $${Q}_{SC}=\frac{S\sigma x(t)}{{d}_{0}+x(t)}$$

10 $${I}_{SC}=\frac{d{Q}_{SC}}{dt}=\frac{S\sigma {d}_{0}}{{({d}_{0}+x\left(t\right))}^{2}}\frac{dx}{dt}=\frac{S\sigma {d}_{0}v}{{({d}_{0}+x\left(t\right))}^{2}}$$

where *v* (m/s) is the moving velocity. Based on the structure of TENG, it can be assumed as a flat-panel capacitor. According to the parallel-plate capacitor model, equivalent capacitance is the combination of three capacitances that can be written as Eq. (11):

11 $$C=\frac{S{\varepsilon }_{0}}{\left(\frac{{d}_{1}}{{\varepsilon }_{r1}}+\frac{{d}_{2}}{{\varepsilon }_{r2}}+x\left(t\right)\right)}$$

*C*(F) is the equivalent capacitor between the two electrodes. To calculate the electric potential distribution in the open circuit (OC) condition, it is assumed that there is no charge transfer and the *Q* is equal to 0. By this assumption, V_OC_ is equal to^56^:

12 $${V}_{OC}=\frac{\sigma x(t)}{{\varepsilon }_{0}}$$

The equations above are used to simulate a flat–flat surface (Ti foil and compact TiO_2_), but to simulate the porous structure in the TENG, the above equations are to be modified to address the electron and mass flows within the porous medium. In the porous layer, the actual surface is different from the nominal surface, so Eq. (7) will be replaced by Eq. (13) based on the model presented by Dharmasena et al.^60,61^:

13 $$V=-\frac{Q}{S{\varepsilon }_{0}}\left(\frac{{d}_{1}}{{\varepsilon }_{r1}}+\frac{{d}_{2}}{{\varepsilon }_{r2}}+x\left(t\right)\right)+\frac{\sigma Ax\left(t\right)}{{S\varepsilon }_{0}}$$

*S* (m^2^) indicates the area of the flat surface of the fixed part and *A* (m^2^) is the real contact area between flat and porous layers. Also in the porous layer in the presence of humidity, the dielectric constant is replaced as below^57^:

14 $${\varepsilon }_{eff}=\frac{1}{4}({\varepsilon }_{s}\left(3{V}_{s}-1\right)+{\varepsilon }_{p}\left(3{V}_{p}-1\right)+{{(({\varepsilon }_{s}\left(3{V}_{s}-1\right)+{\varepsilon }_{p}\left(3{V}_{p}-1\right))}^{2}+8{\varepsilon }_{s}{\varepsilon }_{p})}^{1/2})$$

where ${\varepsilon }_{s}$ and *V*_s_ are dielectric constant and volume fraction of solid and ${\varepsilon }_{p}$ and *V*_p_ are dielectric constant and Volume fraction of pore (air and water) in the porous layer.

Equation (9), (10), and (13) are the basic equations for the contact-mode TENG and can be employed to calculate the electrical output. The numerical parameters and their values are described in Table 1.

**Table 1:** The geometrical parameter in numerical simulation.

Parameter	Symbol (Unit)	value
Thickness of dielectric 1 @ Kapton film	*d*_*1*_ (µm)	20
Thickness of dielectric 2@TiO_2_ nanoparticulated film and nanotube film	*d*_*2*_(µm)	20
Relative dielectric constant of dielectric 1@ Kapton film	${\varepsilon }_{r1}$(–)	3.5
Relative dielectric constant of dielectric 2@ TiO_2_ thin film, nanoparticulate film, and nanotube film	${\varepsilon }_{r2}$(–)	40–140 from refs^62–64^
Nominal surface area	*S*(cm^﻿2^)	2 × 2
Frequency	*f*(Hz)	2
Maximum distance between two electrodes	*x*_*max*_(cm)	2

Layer’s thickness, surface area, frequency, dielectric constant, and induced charge are the numerical simulation inputs. The parameters required for the numerical simulation are listed in Table 1. The presence of humidity in the theoretical model affects two parameters of induced charge and dielectric constant. The dielectric coefficient for compact layers is constant. However, in the porous structure, the fluid layers at high humidity fill the pores making changes the dielectric coefficient. Thus, unlike the flat layers, the porous structure cannot use a constant dielectric coefficient and need to correct by Eq. (14). In the porous layer and in the presence of humidity, the dielectric coefficient is affected by three factors including (1) volume fraction, (2) dielectric constant of TiO_2_ and the fluids (water and air), and (3) porosity. By using Eq. (14), the relationship between the electric charge reduction and humidity is addressed.

## Results and discussions

### Experimental results

**Figure 2:**
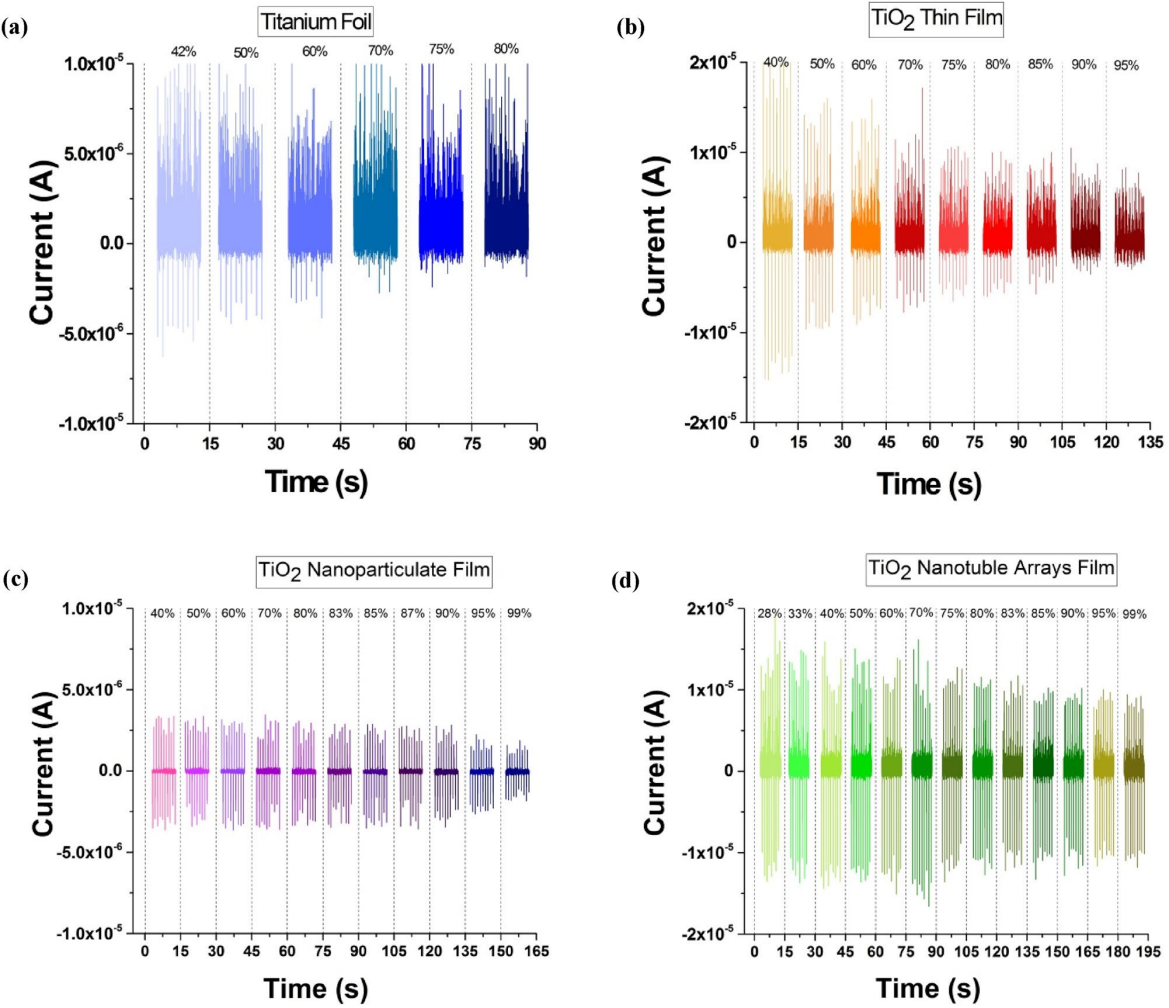
A comparison of the measured current outputs of TENG under various RH between different morphologies of the triboelectric active layer: (**a**) Ti foil, (**b**) TiO_2_ thin film, (**c**) TiO_2_ nanoparticulate film, and (**d**) TiO_2_ nanotube film.

To investigate how surface morphology affects the performance of TENG in humid environments, flat structures consist of Ti foil-TENG and TiO_2_ thin film-TENG were studied firstly. The electrical output current at the different ambient relative humidity is plotted in Fig. 2a,b. Figure 2a shows that the output current decreases with the increase of the ambient humidity. The electrical output current of TENG based on Ti foil starts to decrease from 4.99 ± 0.55 μA to 1.19 ± 0.27 μA when RH increased from 45 to 80%, Which indicates that it has lost 74% of its initial output.

Figure 2b shows the output current of TENG based on the TiO_2_ thin film under various relative humidity conditions. According to the diagram, the measured current at 40% RH has the value of 13.7 ± 1.11 μA, which decreases to 2.65 ± 0.14 μA at 95% RH, respectively.

In the second step, the performance of the TENG based on the porous structure has been investigated, so the TiO_2_ nanoparticulated and nanotube films were selected as the triboelectric active layers according to Fig. 1g,h. The current as a function of RH was measured for TiO_2_ nanoparticulate-TENG and shown in Fig. 2c. Based on Fig. 2c, the output current decreases 53% from 2.91 ± 0.34 μA to 1.34 ± 0.27 μA as the RH increases from 40 to 99%.

Following the study of the effect of surface morphology on TENG performance in a humid environment, TiO_2_ nanotube arrays are used as the active layer of TENG and the output current at different relative humidity is plotted in Fig. 2d. It can be seen that the current-RH diagram has a smooth decreasing trend, so that generated current is reduced from 13.9 ± 2.9 μA at 28% RH to 8.92 ± 0.58 μA at 99% RH.

**Figure 3:**
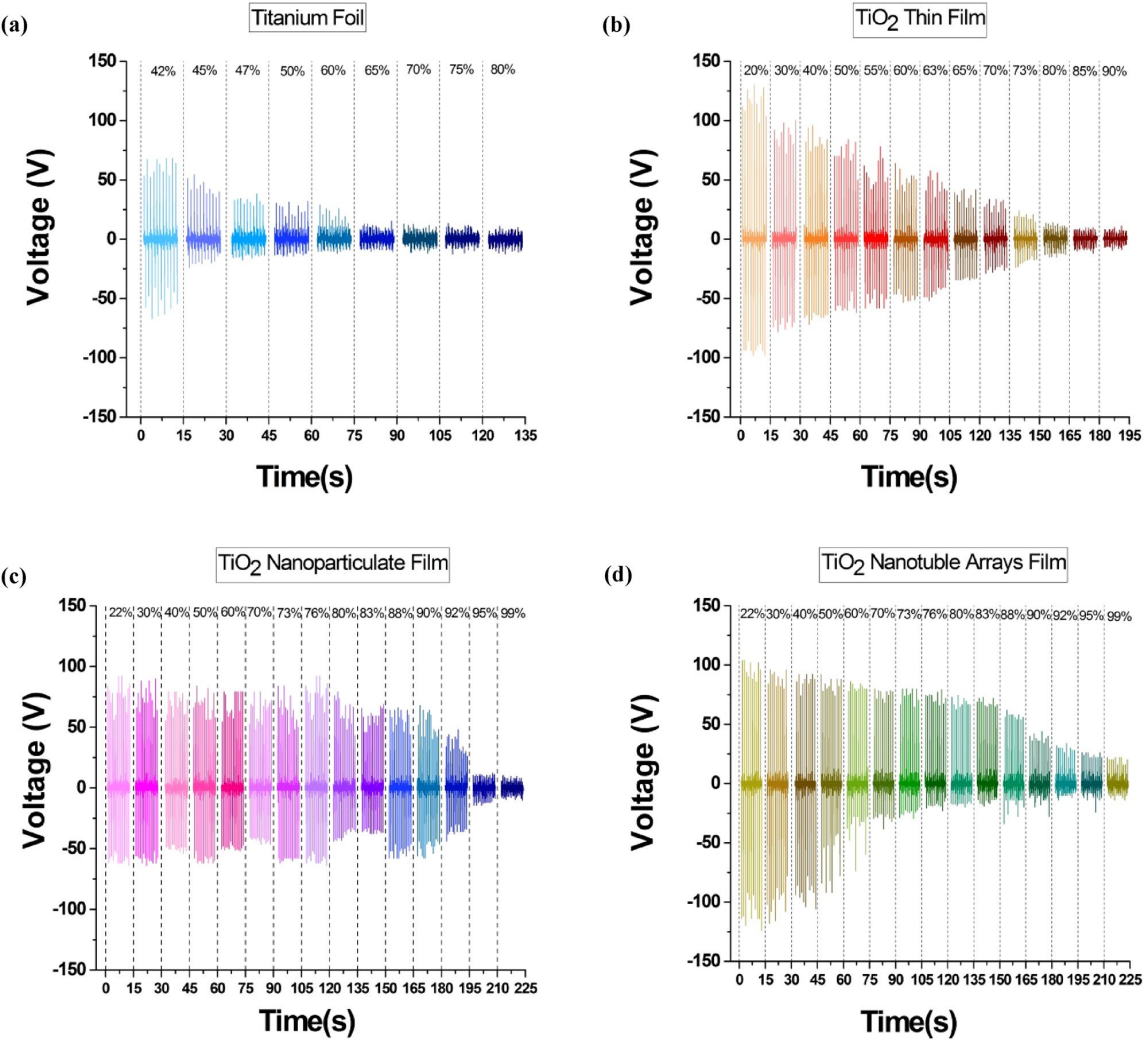
A comparison of the measured voltage outputs of TENG under various RH between different morphologies of the triboelectric active layer: (**a**) Ti foil, (**b**) TiO_2_ thin film, (**c**) TiO_2_ nanoparticulate film, and (**d**) TiO_2_ nanotube film.

The output voltage under various RH is plotted in Fig. 3 for each morphology. It can be seen that the output voltage has the same trend as the current. Based on Fig. 3a, the output voltage of Ti foil-TENG decreases from 43.48 to 8.54 V when the RH increased from 45 to 80%. According to the diagram Fig. 3b, the measured voltage of TiO_2_ thin film-TENG at 40% RH has the values of 76.54 V which decrease to 8.58 V at 95% RH, respectively. Similarly, the output voltage is plotted in Fig. 3c for the TiO_2_ nanoparticulate film-TENG that can be observed that voltage is decreased from 68.96 to 8.48 V by increasing RH from 40 to 99%. Based on Fig. 3d, the generated voltage is reduced from 82.33 V at 28% RH to 19.16 at 99% RH for TiO_2_ nanotube film.

The fluctuation in current and voltage pulse in Figs. 2 and 3 are caused by the factor of rigidity of ceramic layers. Due to the rigidity of ceramic layers, repeatable electron transfer in each tapping may be interrupted since two electrodes cannot reach each other on a microscopic scale. The fluctuation in current and voltage when using ceramic electrodes in triboelectric nanogenerators has also been reported in other published research articles using ceramic electrodes^65–67^.

**Figure 4:**
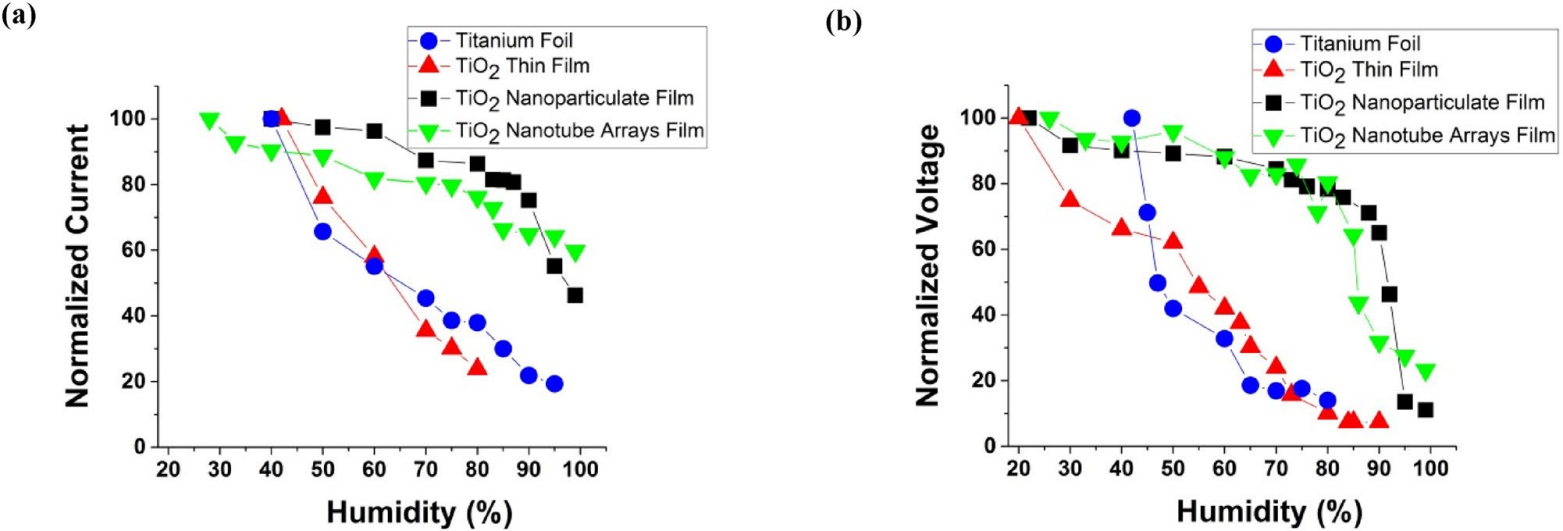
Normalized outputs. A comparison of (**a**) the measured normalized current and (**b**) measured normalized voltage of TENG under various RH between different morphologies of the triboelectric active layer.

To compare the electrical output of four morphologies with each other, the normalized current and voltage are plotted in Fig. 4. According to Fig. 4a,b, it can be seen that the output signals of TENG based on a flat surface (Ti foil and TiO_2_ thin film) decrease linearly with the steep slope by increasing the relative humidity. The reason can be explained that the performance of TENG directly is dependent on the surface charge (*Q*) based on Eqs. (10) and (12). In a humid environment, a water film forms on the triboelectric surface which can decay the generated charges on the surface so cause a decrease in the electrical output of the TENG. By increasing the relative humidity, the amount of water molecules adsorbed on the surface increased, thus caused a severe drop in the output voltage and current at high RH.

In the next case, TiO_2_ nanoparticulate film-TENG is investigated in Fig. 4a,b. The important point is that the electrical current decreases with increasing RH, but this electrical current reduction is nonlinear unlike the TiO_2_ thin film-TENG and Ti foil-TENG. The output electrical current almost is constant up to 80% RH, and by increasing the RH above 80% it starts to drop. TiO_2_ nanoparticulate film has high surface-to-volume on about 40–50 (m^2^/g)^68^ and a porosity of about 50% and because of that it can maintain the current and surface charge up to high RH, unlike a flat surface. By increasing the RH above 80%, the water molecules are adsorbed on the interior surface of the porous media and the electrical output would be decreased.

It can be seen that the normalized current-RH diagram for TiO_2_ nanotube-TENG has a smooth decreasing slope in Fig. 4a. To describe in more detail, as shown in Fig. 2d, the measured current is about 12 μA and was not changed widely as the RH increased from 20 to 80%, then by increasing RH from 80% up to 99%, the output current drops down slowly. One of the reasons is that TiO_2_ nanotubes film has a large interior surface area of about 100–150 (m^2^/g)^68^, which results in increasing electron-trapping capacity in the TENG. Another reason is that at low relative humidity the amount of water molecules is low, and the adsorbed water has little impact on the surface charge, thus TENG can maintain its performance in a humid environment at low RH. By increasing the RH, the water molecules start to adsorbed on the inner and outer layers of the nanotube, so the TENG charge destructs and would be seen a drop in output current at high RH (RH above 80%). The specific surface area is determined with the Brunner-Emmett-Teller (BET) method and is shown in Table 2 for nanotube and nanoparticulate samples.

**Table 2:** The comparison of BET surface area between nanoparticle and nanotube film^68^.

Morphology	BET (m^2^/g)
TiO_2_ nanotube	100–150
TiO_2_ nanoparticulate	40–50

The same trend is observed in output voltage in Fig. 4b. Therefore, as can be seen from Fig. 4a, it can be concluded that the use of a porous structure in the TENG maintains its performance up to high relative humidity, namely up to 80% humidity. To understand this result, the effect of moisture in the TiO_2_ nanoparticulate and nanotube films is investigated from the point of view of physical phenomena in more detail. At a low RH, water molecules are adsorbed on active sites by the chemisorbed processes and the amount of water is not sufficient to cover the internal porous surface, so the electrical outputs are not affected by humidity at low RH and are almost constant. By increasing the RH, water molecules are adsorbed by the physisorbed processes and the amount of molecular layers of water increases which can cover the internal porous surface causing the disappearance of the surface charge leading to decreased electrical output^41,59^. Also, it is clear that the TiO_2_ nanotube electrode can maintain its performance in a humid environment up to RH 80%, similar to TiO_2_ nanoparticulate electrode, but by increasing the RH to 99%, the output current of the TENG based on the TiO_2_ nanotube film is reduced by 40% compared to its own initial state. However, in the TENG based on TiO_2_ nanoparticulate film, the reduction is around 53% when increasing the RH to 99%. Thus, it can be understood that water permeability in TiO_2_ nanoparticles films is higher than the TiO_2_ nanotube film at high RH, thus this shows the important role of the surface morphology.

In a general comparison between the used electrode structures, by increasing RH from 40 to 80% the normalized current decreases 14%, 14% and 62% for TiO_2_ nanotube, TiO_2_ nanoparticulate and Ti foil, respectively; then by increasing RH from 80 to 95% the normalized current decreases 11%, 31% and 18%, respectively. By comparing the results of all four morphologies in Fig. 4, it can be concluded that the electrical output of TiO_2_ nanotube-TENG has good stability under high relative humidity and is suitable in self-powered applications in a humid environment.

**Figure 5:**
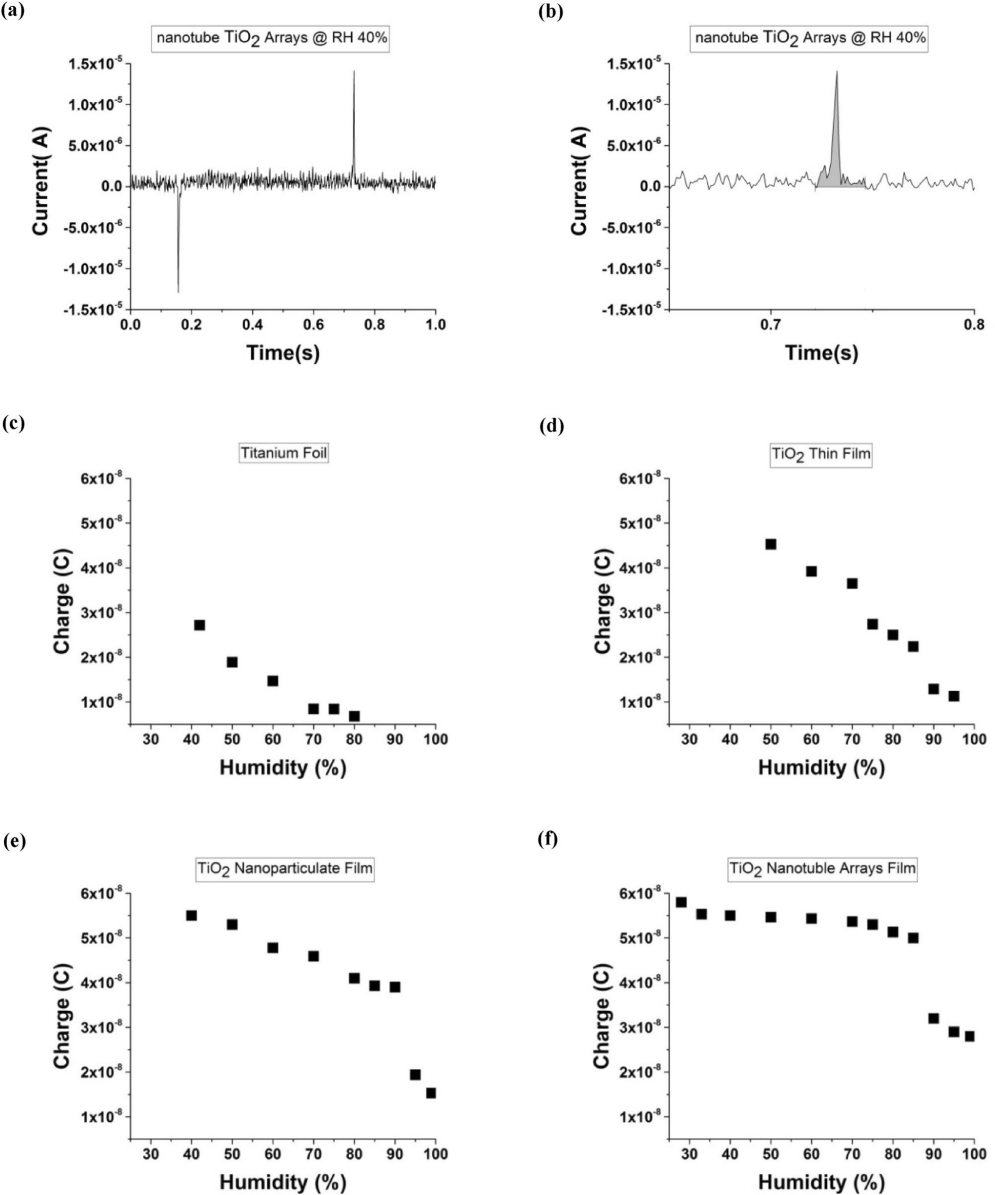
Measured single pulse results and the charge transfer of TENG in different morphology as a function of relative humidity. (**a**) The current at a pressing-releasing mode under a single tap. (**b**) The induced charge based on the integration highlight area under releasing mode. The charge transfer of TENG as a function of relative humidity at: (**c**) Ti foil, (**d**) TiO_2_ thin film, (**e**) TiO_2_ nanoparticulate film, and (**f**) TiO_2_ nanotube film.

Here the amount of induced surface charge is calculated for all four morphologies. The amount of induced charge is equal to the area under the current–time diagram that is obtained by integration of the current *I*(t) over the time interval^69^. Figure 5a,b shows current–time diagram for a single tap and the induced charge is equal to the highlighted area. Figure 5c–f shows charge transfer at various RH for Ti foil, thin film, nanoparticulate, and nanotube active layer, respectively. Similarly, the charge transfer decreases with increasing RH at all electrodes morphology, which confirms water adsorption on the surface and dissipation of the charges. When the RH changes from 40 to 80%, the charge drop is equal to 75%, 66%, 17%, and 7% for Ti foil, TiO_2_ thin film, TiO_2_ nanoparticulate, and TiO_2_ nanotube active layer at the same condition, respectively. It can be seen that the charge drop is lower at TiO_2_ nanoparticulate-TENG and TiO_2_ nanotube-TENG than flat surfaces like TiO_2_ thin film-TENG and Ti foil-TENG structural. The effect of high humidity on electrical charge flow depends on the physical phenomena of water layer formation. The phenomenon is that while a continuous layer of water can be formed on the flat surface, a porous media delays the water layer formation. The porous film has a discontinuous surface and the adsorbed water molecule cannot form a coalescent layer so the surface charge can be stable up to high RH. Also, since the experimental data are measured under the same condition as dielectric thickness, superficial surface area and material, it can be concluded that the use of porous nanostructure due to high real surface area and the process of water permeate on the pore lead to better performance of TENG in humid environments. Also, it can be observed that the variation trend of the output voltage and current at each morphology are consistent with the trend of the induced charge. This observation confirms the direct relationship between the output voltage and current of the TENG with the induced charge that was previously seen in Eqs. (10) and (12).

**Figure 6:**
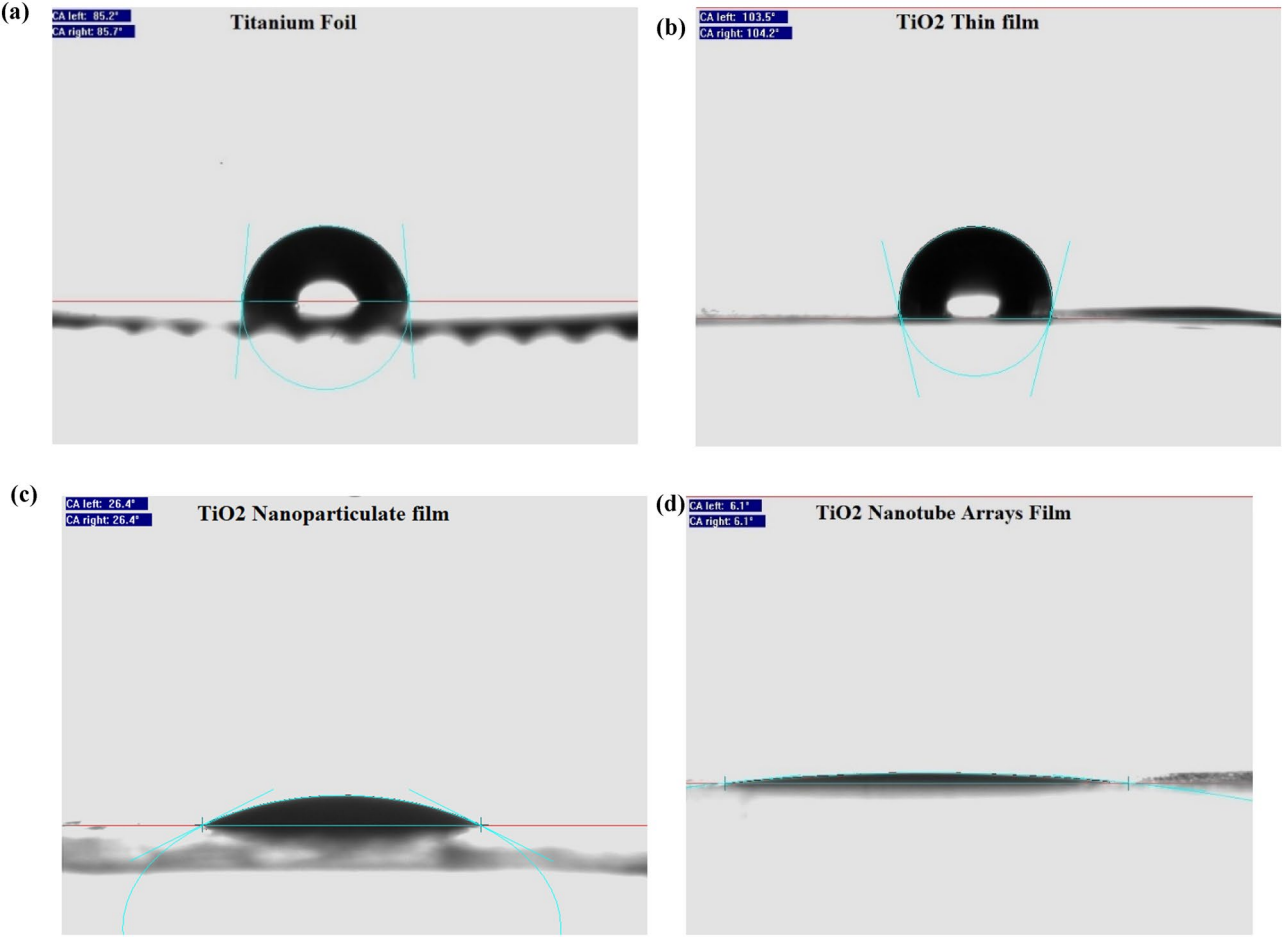
Measurement of the water contact angle on the triboelectric films for (**a**) Ti foil, (**b**) TiO_2_ Thin film, (**c**) TiO_2_ nanoparticulate film, and (**d**) TiO_2_ nanotube film.

Figure 6 shows the measurements of the water contact angle on the triboelectric films for Ti foil, TiO_2_ Thin film, (c) TiO_2_ nanoparticulate film, and (d) TiO_2_ nanotube film. Wettability depends on the morphology, chemical composition and the molecular structure of the surface^42,70,71^. In this work, the influences of the surface morphologies on the wettability of TENG plates were systematically investigated by measuring the water contact angle on the films. Generally, hydrophobicity or hydrophilicity of the surface is obtained by measuring the contact angle between the liquid surface and the outline of the contact surface. The comparisons of the water contact angle on Ti foil, TiO_2_ thin film, TiO_2_ nanoparticle, and TiO_2_ nanotube surfaces are shown in Fig. 6a–d. The contact angle on the Ti foil is about 85°. Based on Fig. 6, as the Ti foil became oxidized, the hydrophobicity of the Ti layer is increased by enhancement of contact angle from 85° for Ti foil to 103° for TiO_2_ thin film. The orientation of water molecules makes the TiO_2_ thin film a hydrophobic surface. The hydrophobicity of flat TiO_2_ film would be related to the orientation of interfacial water molecules. However, the origin of the hydrophobicity of the flat TiO_2_ film is still unclear^72^. TiO_2_ nanotube arrays and nanoparticle palates show hydrophilic behavior. The contact angles are 26° and 6° for the TiO_2_ nanoparticle and TiO_2_ nanotube, respectively. It can be seen that nanotube structures made the surface hydrophilic. This may be due to the hydroxide compounds on the nanotube surfaces. Also, TiO_2_ arrays morphology provides that the liquid penetrates and in addition decrease the contact angle making a hydrophilic surface^72,73^.

### Numerical results

In the next step, the TENGs are simulated by using the numerical model developed within this work. This developed model clarifies the relationship between the electrical charge drop and humidity.

The calculated induced charge can be seen in Fig. 5. By substitute input parameters in Eq. (10) to Eq. (14), the output current and voltage are obtained by employing the modeled governing contact-separation mode TENG, and then the results are compared with the experimental data.

**Figure 7:**
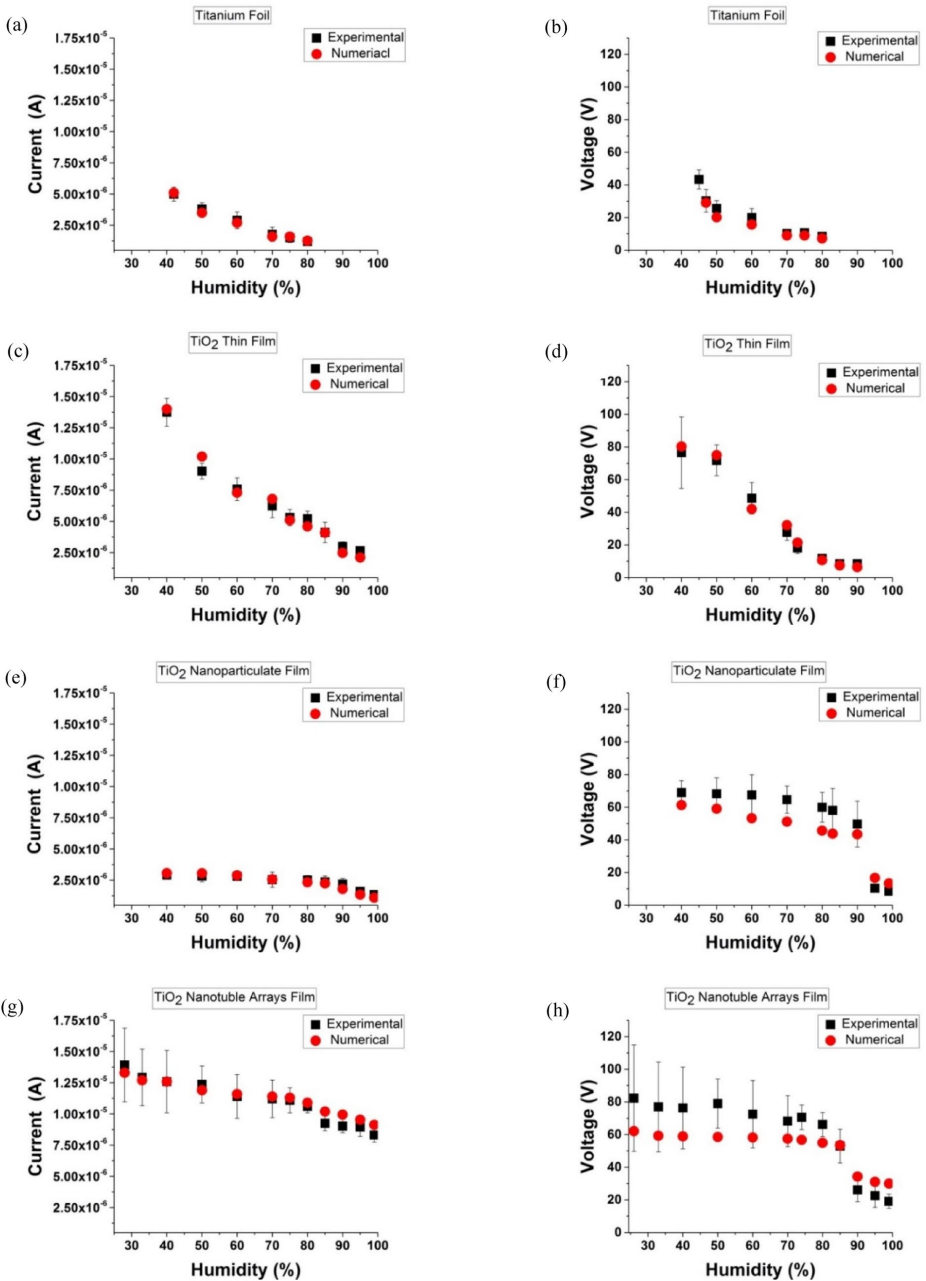
Comparison between the simulated electrical output and the experimental data in different morphologies of the triboelectric active layer: (**a,b**) Ti foil, (**c,d**) TiO_2_ thin film, (**e,f**) TiO_2_ nanoparticulate film, and (**g,h**) TiO_2_ nanotube film.

Ti foil-TENG is a metal-to-dielectric contact-mode TENG and the capacitance is calculated according to the thickness and dielectric constant of the Kapton film in Eq. (11). Figure 7a,b shows the output current and voltage for the Ti foil-TENG base on numerical simulation. It can be observed that the results have a good agreement with the experimental data and the output decrease linearly by increasing RH.

TiO_2_ thin film-TENG is a dielectric-to-dielectric contact-mode TENG and the capacitance can be determined through the thickness and dielectric constant of the Kapton and TiO_2_ films in Eq. (11). The numerical outputs compared with the experimental data at various RH for TiO_2_ thin-film TENG and had a good agreement with it (Fig. 7c,d). Also, it can be observed that the results drop linearly like Ti foil. However, the output of the TiO_2_ thin film is higher than that in the Ti foil electrode, in the same condition because of different equivalent capacitors between the metal-dielectric and dielectric-dielectric modes of TENG.

Figure 7e,f shows the numerical results of the TiO_2_ nanoparticulate case, which follow the trends of the experimental results. The numerical output of the TiO_2_ nanotube film is presented in Fig. 7g,h and confirmed the experimental model results. The dropping part of electrical output in the nanoparticulate and nanotube film at the numerical modeling is due to two effects: firstly, dropping the surface charge, and secondly, the change in the effective dielectric constant in Eqs. (10) and (12). By permeation of the water molecules in the porous structure, water replaces the air in the pores leading to the change in the effective dielectric constant is changed.

**Figure 8:**
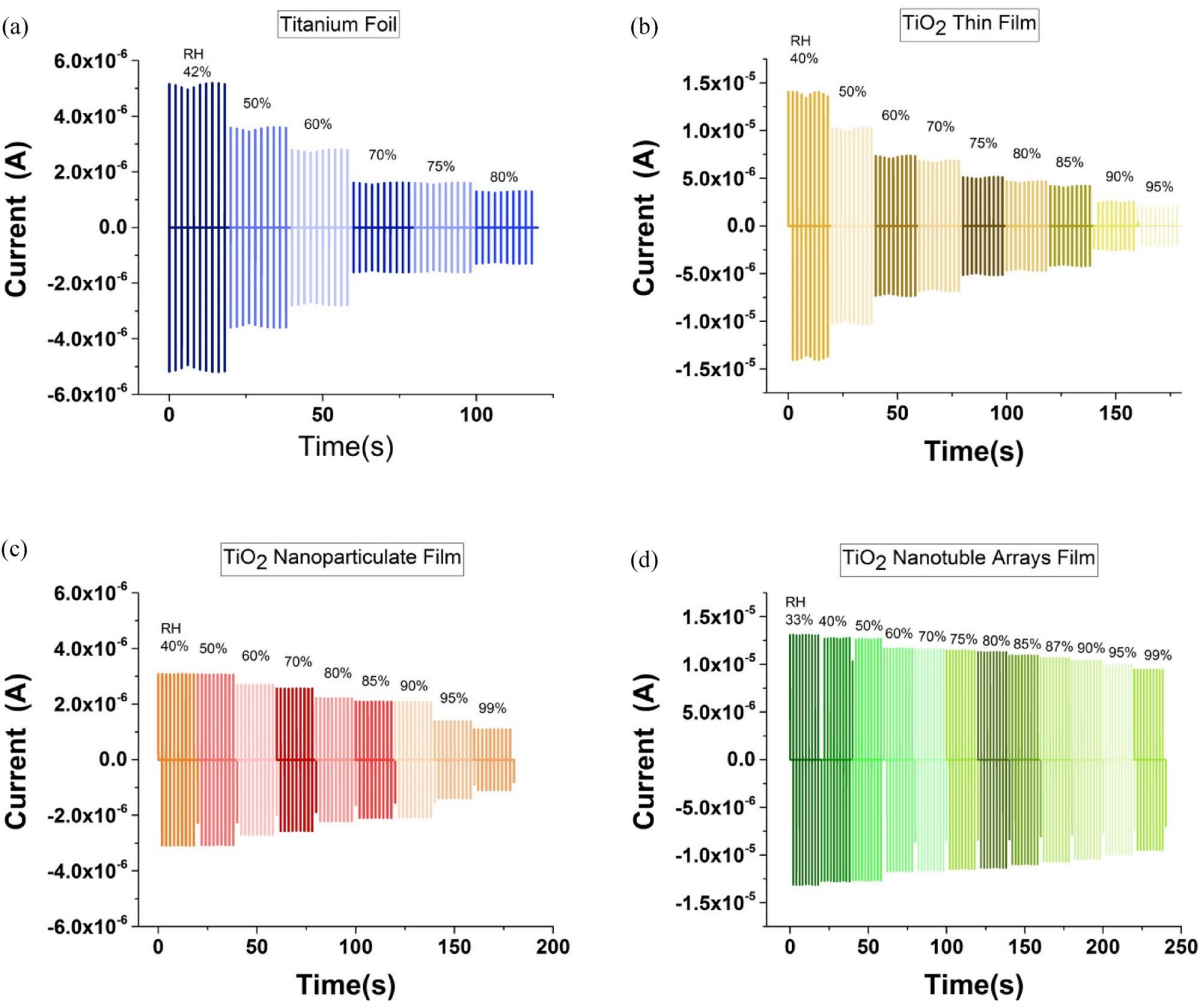
Simulated pulse current for (**a**) Ti foil, (**b**) TiO_﻿2_ thin film, (**c**) TiO_2_ nanoparticulate film, and (**d**) TiO_2_ naotube film at different RH.

The pulse current was calculated numerically at different RH and compared in Fig. 8a-d for Ti foil, TiO_2_ thin film, TiO_2_ nanoparticulate and TiO_2_ nanotube TENG. At each specific RH, the pulse is obtained by using *Q* from Fig. 5 and Eq. (10). Figure 8 displays that the TiO_2_ nanotube-TENG can maintain its quality up to high RH compared with other morphologies due to the limited moisture permeation. Thus, one can conclude that among the four studied morphologies, TiO_2_ nanotube is the best choice for TENG application and self-powered active sensors at high humidities.

## Conclusion

In this study, the effect of surface morphology on the TENG performance investigated under various relative humidities. Four morphologies based on Ti foil, TiO_2_ thin film, TiO_2_ nanoparticulate film, and TiO_2_ nanotube film were used as triboelectric active layers. The electrical output was obtained experimentally and numerically in all morphologies. A mathematical method developed to model the triboelectric nanogenerators showing good agreement with measurements. The results show that by increasing the RH, the electrical output of TENG has a significant drop at the flat surface but by using the nanoparticle and nanotube structural, TENG can maintain its quality and performance up to high RH. Thus, the electrical outputs of flat structures such as Ti foil and TiO_2_ thin film decrease to 50% of the initial performance at RH about 50%, while in nanostructured porous films such as nanotube and nanoparticle films 50% electric output drop occurs at RH above 95%. This is due to the limitation of the water permeation in the porous structure that delays water layer formation in porous structures, unlike the flat structure where a continuous layer of water form on the surface and prevent the charge. To study the reasons for water penetration in the surface, measurements of the surface wettability are performed in this work showing that the nanotubes and nanoparticle films have contact angles of about 6° and 26° respectively representing hydrophilic surfaces causing water to penetrate into the pores. However, the flat structures show a hydrophobic property on their surface forcing the formation of water films on the surface preventing electron transfer and consequently reducing the power output at high humidities. Our results indicate that utilizing nanostructured electrodes can enhance the performance of TENGs as power generators as well as self-powered active sensors in harsh humidity conditions.
